# Antidepressant Medication Use and Risk of Hyperglycemia and Diabetes Mellitus—A Noncausal Association?

**DOI:** 10.1016/j.biopsych.2011.07.008

**Published:** 2011-11-15

**Authors:** Mika Kivimäki, G. David Batty, Markus Jokela, Klaus P. Ebmeier, Jussi Vahtera, Marianna Virtanen, Eric J. Brunner, Adam G. Tabak, Daniel R. Witte, Meena Kumari, Archana Singh-Manoux, Mark Hamer

**Affiliations:** a Department of Epidemiology and Public Health, University College London, London, United Kingdom; b Institute of Behavioural Sciences, University of Helsinki, Helsinki, Finland; c Department of Psychiatry, University of Oxford, Warneford Hospital, Oxford, United Kingdom; d Finnish Institute of Occupational Health, Helsinki, Finland; e University of Turku, Turku University Hospital, Turku, Finland; f First Department of Medicine, Faculty of Medicine, Semmelweis University, Budapest, Hungary; g Steno Diabetes Center, Gentofte, Denmark; h Institut National de la Santé et de la Recherche Médicale, Paris, France

**Keywords:** Antidepressants, depression, drug toxicity, glucose, pharmacotherapy, type 2 diabetes mellitus

## Abstract

**Background:**

Previous research suggests a link between antidepressant use and diabetes, but it is unclear whether the association is causal or attributable to detection/ascertainment bias. To examine this, we assessed the associations of antidepressant use with change in glucose levels and incidence of undiagnosed and diagnosed diabetes.

**Methods:**

During an 18-year period, we monitored antidepressant use, glucose levels, and diabetes status in 5978 civil servants (70.9% male, age range 39–64 years) free of diabetes at baseline (the Whitehall II study). Use of medication and plasma glucose were assessed at four study screenings: 1991/1993, 1997/1999, 2003/2004, and 2008/2009. Incident diabetes cases were classified as either diagnosed (*n* = 294) if detected using self-report of physician diagnosis and/or the use of diabetes medication or undiagnosed (*n* = 346) if detected based on fasting and/or 2-hour postload glucose levels using an oral glucose tolerance test at the study screenings.

**Results:**

Incidence of diagnosed diabetes was higher among antidepressant users than nonusers (odds ratio 3.10, 95% confidence interval: 1.66–5.78). However, antidepressant use was not associated with undiagnosed diabetes at any follow-up examination nor with higher fasting or 2-hour postload plasma glucose levels or increasing glucose levels over time. Odds ratio for undiagnosed diabetes for antidepressant users versus nonusers was .88 (95% confidence interval: .45–1.72, *p* = .70). The mean difference in glucose changes between participants reporting antidepressant use at three screenings compared with those not on antidepressant treatment was .0 mmol/L.

**Conclusions:**

The link between antidepressant use and diabetes risk may not be causal in nature.

Antidepressants are among the most prescribed drugs worldwide (1,2). Recent large-scale studies based on medical records have linked antidepressant medication use with type 2 diabetes, raising the concern that persistent use of antidepressants might increase the risk of diabetes (3–10). However, these findings should be interpreted with caution. First, the association may be due to indication bias (11); that is, the true association may not be between the medication and the outcome but between the indication for the medication and the outcome, in this case between depression and incidence of diabetes. Second, detection/ascertainment bias is possible as depressed patients on antidepressant treatment may use health services more often than untreated patients or nondepressed people, increasing their likelihood of being diagnosed with medical conditions such as diabetes (12). Third, the relation between depression and diabetes is complex, potentially bidirectional, and it is also likely to reflect common antecedent causes, such as obesity, socioeconomic status, and lifestyle factors (13–15). This may confound observed associations between antidepressant use and diabetes.

In the present study, we used data from the British Whitehall II longitudinal study to evaluate the relation between antidepressant use and diabetes. We examined the strength of this association in physician-diagnosed diabetes (diagnosis made by health care provider before the Whitehall II study screening) as compared with study screen-detected diabetes (diabetes detected for the first time by routine blood testing as part of the Whitehall II study). Crucially, robust and equally strong associations with physician-diagnosed and screen-detected diabetes would provide evidence against detection/ascertainment bias. We also examined the association between antidepressant medication use and subsequent change in blood glucose levels in nondiabetic persons. If antidepressant use increases diabetes risk, then nondiabetic antidepressant users would show greater increases in fasting and/or postload glucose concentrations compared with nonusers.

## Methods and Materials

### Study Population

**Figure 1:**
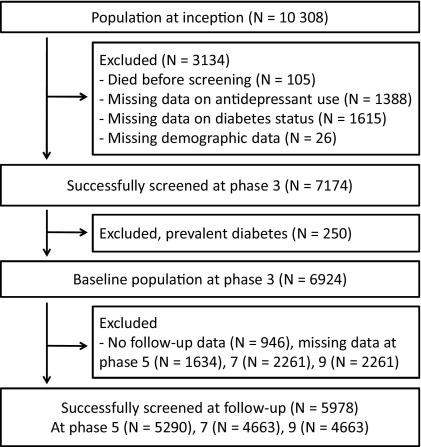
Study flow diagram for diabetes analyses.

We used data from the British Whitehall II study of 10,308 civil servants aged 35 to 55 years at recruitment in 1985/1988 (16). In the 1991/1993 sweep of data collection (phase 3), participants underwent an oral glucose tolerance test (OGTT) for the first time. This phase forms the baseline for the analyses we report here. Clinical examination with OGTT was repeated on three subsequent occasions, in 1997/1999 (phase 5), 2003/2004 (phase 7), and 2008/2009 (phase 9) (17). A total of 5978 men and women without diabetes at baseline participated at least in one of the three follow-up examinations and formed the analytic sample of the present study (Figure 1). The University College London ethics committee reviewed and approved the study; written informed consent was obtained from each participant at each clinical examination.

### Assessment of Clinical Characteristics

Demographic characteristics at each examination were age, sex, ethnicity (White vs. non-White), and socioeconomic status, defined by the current or most recent employment grade in the British civil service, divided into three categories (high = administrative; medium = professional or executive, and low = clerical or support).

At each clinical examination, body mass index (weight [kg]/height squared [m^2^]), waist circumference, and systolic blood pressure were measured using standardized protocols (18,19). On the fasting samples, lipid profile, including assessments of high-density lipoprotein cholesterol and triglycerides, were analyzed as previously described (20). Use of antihypertensive and lipid-lowering medication and smoking status (current smoker vs. nonsmoker) were requested. Physical activity was assessed based on response to questions on the frequency and duration of participation in moderately energetic (e.g., dancing, cycling), and vigorous physical activity (e.g., running, playing squash). Participants were classified as inactive (<1 hour/week of moderate physical activity and <1 hour/week of vigorous physical activity) versus other. Alcohol consumption in the previous week was measured as units per week. Severity of depressive symptoms, assessed only in 2003/2004, was defined based on the summary score of the Center for Epidemiologic Studies Depression Scale (21), a measure that has been validated among diabetic patients (22).

### Assessment of Antidepressant Medication Use and Type 2 Diabetes

At each screening, participants provided details of current medications use (generic name, brand name, or both); these were subsequently coded using the British National Formulary to determine antidepressant use (23). At each screening, blood was drawn after at least 5 hours of fasting. An OGTT involved study members drinking a 75 g glucose solution, 2 hours after which venous blood was again taken. Blood glucose was measured using the glucose oxidase method (24) on a YSI MODEL 2300 STAT PLUS Analyzer (YSI Corporation, Yellow Springs, Ohio) (mean coefficient of variation: 1.4%–3.1%) (25). Type 2 diabetes was defined as fasting glucose ≥7.0 mmol/L or a 2-hour postload glucose ≥11.1 mmol/L during the OGTT performed at the Whitehall screening and as physician-diagnosed diabetes or use of diabetes medication for those with diagnosed diabetes (26). At each screening, diabetes cases were classified as physician-diagnosed if their clinical diagnosis was already known (i.e., they reported that their family physician had diagnosed diabetes and/or prescribed antidiabetic medication) or as study screen-detected if their diagnosis was first made by abnormal fasting or 2-hour postload glucose levels during the Whitehall II clinical screening.

In the Whitehall II results letter sent to all study participants, we reported abnormal results (including raised glucose) and advised the participants to contact their general practitioner, but we neither screened for clinical depression nor assessed the need for antidepressant therapy.

### Statistical Analysis

All statistical analyses were performed using STATA 11.0 software (StataCorp LP, College Station, Texas). Statistical tests were two-sided; a *p* value of less than .05 was considered statistically significant. Differences in baseline characteristics between study participants treated with antidepressants at any of the four clinical screenings and those never on antidepressants (irrespective of diabetes during the follow-up) were tested by using chi-square test and analysis of variance, as appropriate.

In all analyses, diabetes (irrespective of physician-diagnosed or screen-detected) was considered only at the first occurrence and coded as missing value at subsequent phases. We used logistic regression analysis to examine the age-, sex-, and ethnicity-adjusted associations of antidepressant use at baseline (phase 3) with incident physician-diagnosed diabetes and incident study screen-detected diabetes at any of the phases 5, 7, or 9. To obtain an estimate of the cross-sectional associations between time-dependent measures of antidepressant use and incident physician-diagnosed and study screen-detected diabetes at phases 5, 7, and 9, we used multilevel logistic regression with the generalized estimating equations method (Supplement 1 gives details for this method).

To examine biological plausibility (i.e., the association between antidepressant use and change in glucose levels), we plotted unadjusted means of fasting and 2-hour plasma glucose at each study phase by status of antidepressant use and fitted age-, sex-, and ethnicity-adjusted linear trends in glucose levels across the study phases among participants not diagnosed as diabetic by a physician (trends were obtained using multilevel linear regression with participant as the clustering factor and study phase as the time variable). We used linear regression to determine whether antidepressant use at each clinical screening predicted subsequent change in fasting and 2-hour plasma glucose levels between that and the following examination. We performed multilevel linear regression analyses, with generalized estimating equations, to obtain a combined estimate for these associations across the phases (i.e., antidepressant use at phase 3 predicting change in glucose between phases 3 and 5, antidepressant use at phase 5 predicting glucose change between phases 5 and 7, and antidepressant use at phase 7 predicting change in glucose between phases 7 and 9) (Supplement 1 gives details for this method). We ran a corresponding analysis for the association between length of exposure to antidepressant use (defined as the number of times reported antidepressant use at the current and preceding clinical examinations) and subsequent change in glucose levels (see Supplement 1 for details).

To examine potential confounding, we repeated the analyses with multiple clinical characteristics at each phase added as covariates in the model. As a test of reverse causation, we performed a logistic regression on diabetes status at baseline as a predictor of antidepressant use at follow-up among participants not on antidepressant treatment at baseline.

## Results

Of the 5978 participants, 70.9% (*n* = 4238) were men, 92.0% (*n* = 5501) were White, and 1.6% (*n* = 94) were treated with antidepressants at study baseline. Mean age at baseline was 49.2 (range 39–64) years. These figures were very similar among all 6924 nondiabetic participants at baseline (70.0% male, 91.4% White, mean age 49.3 years, prevalence of antidepressant users 1.7%), as well as among all 7174 successfully screened participants (69.9% male, 90.7% White, mean age 49.4 years, prevalence of antidepressant users 1.7%) (Figure 1).

Table 1 shows baseline characteristics for study participants treated with antidepressants at any of the four clinical screenings (7.0%, *n* = 419) as compared with those not on antidepressants. In addition to depressive symptoms (measured at phase 7), antidepressant use was associated with female sex, lower occupational position, more frequent antihypertensive medication use and sedentary lifestyle, higher prevalence of smoking, and slightly smaller waist circumference.

### Diabetes Risk

We identified 346 study screen-detected diabetes cases in one of the three follow-up screenings; 294 were diagnosed by their physician before the screening. Use of antidepressants at baseline was not associated with incident study screen-detected diabetes but a strong association was observed with incident physician-diagnosed diabetes (age-, sex-, and ethnicity-adjusted odds ratio 3.10, 95% confidence interval [CI]: 1.66, 5.78; Table 2). There was no statistical evidence of sex differences in these associations (*p* values for sex interaction .22 and .80 in models containing the main effects).

Table 3 shows cross-sectional associations between antidepressant use and incident diabetes over the repeated measurements. Again, no association with study screen-detected diabetes (adjusted odds ratio across phases 5, 7, and 9: .88, 95% CI: .45, 1.72) and a strong association with physician-diagnosed diabetes (odds ratio 2.34, 95% CI: 1.46, 3.75) were observed (for phase-specific associations, see Table S1 in Supplement 1).

### Change in Plasma Glucose Levels

**Figure 2:**
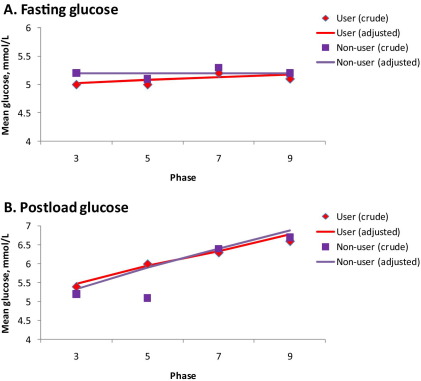
Mean fasting plasma glucose **(A)** (*n* = 5487) and 2-hour postload plasma glucose **(B)** (*n* = 4991) by study phase and antidepressant use. Linear trends are adjusted for age, sex, and ethnicity.

In further analyses, participants with physician-diagnosed diabetes at any screening were excluded from the analysis. Data for fasting and 2-hour plasma glucose levels were available for 5487 and 4991 participants, respectively. As shown in Figure 2, unadjusted mean plasma glucose levels at each study phase were similar for antidepressant users and nonusers. In addition, no difference was seen in age-, sex-, and ethnicity-adjusted trends over time in fasting glucose (*p* values for antidepressant and antidepressant × time interaction terms .26 and .11, respectively) or 2-hour postload glucose (*p* values .37 and .32) between the groups. A corresponding within-person change analysis fitted with fixed-effect estimator replicated this finding (*p* values .12 and .18 for fasting glucose and .77 and .76 for postload glucose).

Similarly, neither status of antidepressant use nor length of exposure to antidepressant use was associated with subsequent change in fasting or 2-hour glucose (Table 4; for phase-specific analysis, see Table S2 in Supplement 1). For example, the mean difference in fasting and 2-hour postload glucose changes between participants reporting antidepressant use at three examinations compared with those not on antidepressants was .0 mmol/L.

### Analysis of Confounding and Reverse Causation

The association between antidepressant use and physician-diagnosed diabetes was little changed after multivariable adjustment for age, sex, ethnicity, socioeconomic status, body mass index, waist circumference, systolic blood pressure, high-density lipoprotein cholesterol, triglycerides, antihypertensive and lipid-lowering medications, smoking, physical activity, and alcohol consumption (Table S3 in Supplement 1). Similarly, the absence of association between antidepressant use, study screen-detected diabetes, and plasma glucose remained unchanged after the adjustment (Table S3 in Supplement 1), as well as in a complete case analysis of participants with no missing data at any study phase (Table S4 in Supplement 1).

There was no evidence to suggest that depressive symptoms explained the lack of association between antidepressant use and study screen-detected diabetes (odds ratios from a model adjusted for depressive symptoms in addition to age, sex, and ethnicity .76, 95% CI: .23–2.46, *p* = .65; and .60, 95% CI: .22–1.65, *p* = .32 at phases 7 and 9, respectively) because controlling for depressive symptoms did not increase the magnitude of this association. Depressive symptoms were strongly associated with antidepressant use (age-, sex-, and ethnicity-adjusted odds ratio 6.30, 95% CI: 4.43–9.00, *p* < .0001 at phase 7 and 5.50, 95% CI: 4.02–7.52, *p* < .0001 at phase 9) and physician-diagnosed diabetes (odds ratio 1.67, 95% CI: 1.05–2.65, *p* = .03 at phase 7 and 1.64, 95% CI: 1.20–2.23, *p* = .002 at phase 9).

We found support for the notion of reverse causation (i.e., a longitudinal association from physician-diagnosed diabetes in relation to later antidepressant use) in an analysis of 6541 participants who reported not taking antidepressant treatment at baseline (for sample selection, see Figure S1 in Supplement 1). Of them, 5.5% (*n* = 361) began antidepressant treatment during study follow-up and the proportion was higher among participants with physician-diagnosed diabetes at baseline than in others (age-, sex-, and ethnicity-adjusted odds ratio 1.72, 95% CI: 1.02–2.88, *p* = .04).

## Discussion

In this prospective cohort study of almost 6000 middle-aged men and women, we found antidepressant use at four clinical examinations over an 18-year period to be associated with physician-diagnosed diabetes. However, we observed no association between antidepressant use and study screen-detected diabetes; that is, diabetes detected for the first time by routine blood testing as part of the Whitehall II study. Furthermore, there was no association between antidepressant use and glucose levels at any of the four clinical examinations, and continued antidepressant use was not associated with progressively increasing levels of fasting or 2-hour postload glucose over time. These data suggest the observed associations between antidepressant therapy and increased risk of diabetes are not causal.

Our findings on physician-diagnosed diabetes are in agreement with the majority of register-based investigations showing a link between long-term antidepressant use and a clinical record of diagnosed diabetes (3–8,27,28). However, as register-based studies do not capture people with undiagnosed diabetes, such evidence is potentially affected by detection/ascertainment bias (11). First, the observed higher proportion of physician-diagnosed diabetes cases among antidepressant users may relate to the indications for this drug treatment; that is, diabetes may be detected when ruling out endocrinologic diseases as a cause of depression (29). Second, antidepressant use requires contact with medical care, which may increase the likelihood of the diagnosis of hidden health problems such as diabetes (8). These explanations are consistent with the observed differential association with undiagnosed (study screen-detected) and diagnosed (physician-diagnosed) diabetes and the failure to observe a relationship between antidepressant use and undiagnosed diabetes. Our findings are also in agreement with trials on antidepressant medication that do not indicate excess short-term risk of type 2 diabetes (30,31).

Unlike clinical record studies, the Diabetes Prevention Program trial targeted people who were at high risk of diabetes because of overweight and elevated blood glucose levels (10). In that study, participants were randomly assigned to groups of lifestyle changes, glucose-lowering medication (metformin), or placebo (10). The authors found that in the lifestyle and placebo groups, participants consistently on antidepressants during the study period were about twice as likely as nonusers to develop diabetes, although no such pattern was seen in the metformin group (10). However, the study did not report stratified analyses for physician-diagnosed versus study screen-detected diabetes or comparisons of glucose trajectories between antidepressant users and nonusers. Furthermore, despite being based on a clinical trial, the analyses of antidepressant use in the Diabetes Prevention Program utilized observational data because the exposure of interest, antidepressant use, was not randomized in that study (10). Thus, the observed association between antidepressant use and diabetes might have been due to unmeasured differences between the two groups of antidepressant use rather than a causal effect of antidepressant use.

Several potential confounding factors for the association between antidepressant use and diabetes have been hypothesized. For example, antidepressant use could be a proxy of more severe depression or a history of chronic or recurrent depression, which are robust predictors of type 2 diabetes, independent of antidepressant therapy (14,29,32). In the present study, antidepressant users were also more sedentary at baseline and had a higher prevalence of smoking as compared with nonusers. In addition, antidepressant users were more likely to come from low socioeconomic groups, a predictor of both depression and diabetes. However, adjustment for these factors did not change the association between antidepressant use and physician-diagnosed diabetes. Our study, in combination with the evidence that physician-diagnosed diabetes also predicts future antidepressant use (33), is consistent with a view that being treated for one condition increases the likelihood of being diagnosed with the other condition, irrespective of other characteristics of the patient.

Confounding factors may inflate, but could also suppress, the magnitude of an unadjusted association, contributing to false null findings. In the present study, antidepressant use was unrelated to screen-detected diabetes and plasma glucose both before and after adjustments for potential confounding factors, suggesting that the absence of associations with undiagnosed diabetes and glucose is not an artifact resulting from a suppression effect of the confounders.

It is important to consider potential limitations to the present study that could contribute to false-negative findings. First, the participants of the Whitehall II study are from an occupational cohort that is likely to cover a more restricted range of health status compared with the general population. However, a large bias due to restricted variance seems unlikely because the magnitude of the association between antidepressant use and diagnosed diabetes was comparable with that observed in other cohorts (3–10). Second, despite a high response to the successive data collection phases, loss to follow-up accumulated over the extended time period, as is inevitable in all long-term prospective studies. However, differences between the included participants and the total baseline population were generally small. Third, despite the large sample size, the number of diabetes cases among antidepressant users was relatively small; thus, the findings should be interpreted with caution. We did not have precise information on prescriptions (e.g., dosage) and the sample size was not large enough for analyses of specific classes of antidepressants. Given that side effects may vary depending on a drug's chemical substance, antidepressant-specific analyses should be undertaken in future studies (34). Fourth, error in the measurement of glucose and diabetes status is a potential source of false-negative findings. This seems, however, an unlikely explanation of our findings because we used the World Health Organization diabetes definition, based on standard oral glucose tolerance testing, considered to be a gold standard measure (26). Indeed, few previous studies have data based on glucose test available across repeated examinations. Fifth, we identified persons with depressive symptoms using a standard, validated questionnaire measure: the Center for Epidemiologic Studies Depression Scale (21,22). This instrument has been shown to be a sensitive measure of mental health problems in the general population and in diabetic patients but was not designed to make a psychiatric diagnosis of first or recurrent major depression. Thus, we cannot exclude the possibility of confounding by unmeasured depression. However, as depression is known to underlie antidepressant use and increased risk of diabetes, unadjusted associations between antidepressant use and diabetes will represent, if anything, overestimates rather than underestimates of the true association. It therefore seems unlikely that unmeasured depression removed the associations of antidepressant use with screen-detected diabetes and glycemia in our study, as indication bias by depression should have inflated these associations.

### Conclusion

We have demonstrated that detection/ascertainment bias may have compromised evidence in this field of research. Our longitudinal study of British men and women suggests that the adverse effect of antidepressant use on type 2 diabetes risk is biologically implausible and might have been overestimated in previous epidemiologic studies. This evidence suggests that concerns about important diabetogenic side effects of antidepressants might have been unfounded.

## Figures and Tables

**Table 1:** Baseline Characteristics of the Study Population by Antidepressant Medication Use, the Whitehall II Study (*n* = 5978)

Baseline Characteristic^a^	Never Users^b^		Ever Users^b^		*p* Value
	Number of Participants	Mean (SD) or %	Number of Participants	Mean (SD) or %	
Mean Age, Years	5559	49.2 (6.0)	419	48.9 (5.8)	.24
Sex, % Male	5559	71.9	419	58.2	<.0001
Ethnicity, % White	5559	91.8	419	94.5	.051
Occupational Position, % High	5524	42.1	419	32.5	.001
Body Mass Index, kg/m^2^	5524	25.1 (3.5)	418	25.1 (3.8)	.96
Waist Circumference, cm	5462	85.6 (11.3)	414	84.2 (12.7)	.01
Systolic Blood Pressure, mm Hg	5527	119 (13)	419	119 (13)	.06
HDL Cholesterol, mmol/L	5515	1.4 (.4)	418	1.5 (.4)	.11
Triglycerides, mmol/L	5532	1.4 (1.1)	419	1.4 (1.0)	.53
Antihypertensive Medication, %	5559	5.4	419	8.6	.006
Lipid-Lowering Medication, %	5559	.7	419	.1	.49
Current Smoking, %	5524	11.9	419	16.0	.01
Alcohol Consumption, Units per Week	5520	10.6 (12.6)	418	10.1 (12.9)	.39
Sedentary Lifestyle, %	5526	17.6	419	26.5	<.0001
Depressive Symptoms, % c	4503	12.5	340	37.7	<.0001

HDL, high-density lipoprotein; SD, standard deviation.

a Baseline refers to phase 3 (1991/1993) in the Whitehall II study. The numbers in characteristic slightly vary due to missing values.

b Antidepressant use was assessed at phases 3, 5 (1997/1999), 7 (2003/2004), and 9 (2008/2009).

c Based on Center for Epidemiologic Studies Depression Scale score >16 at phase 7.

**Table 2:** Status of Antidepressant Use at Baseline and Incident Diabetes Mellitus at Follow-up by Method of Detection^a^

	Number of Participants (Number of Diabetes Cases)	Odds Ratio	95% Confidence Interval	*p* Value
Antidepressant Use at Phase 3		Outcome: Incident physician-diagnosed diabetes		
No	5884 (282)	1.00		
Yes	94 (12)	3.10	(1.66 to 5.78)	<.0001
Antidepressant Use at Phase 3		Outcome: Incident study screen-detected diabetes		
No	5884 (340)	1.00		
Yes	94 (6)	1.24	(.54 to 2.87)	.62

a Baseline refers to phase 3 (1991/1993) in the Whitehall II study. Follow-up refers to phases 5 (1997/1999), 7 (2003/2004), and 9 (2008/2009). All participants were free of diabetes at baseline. Odds ratios for incident diabetes are adjusted for age, sex, and ethnicity.

**Table 3:** Cross-Sectional Association Between Status of Antidepressant Use and Diabetes Mellitus by Method of Detection^a^

Phase Antidepressant Use	Number of Observations (Number of Diabetes Cases)	Odds Ratio (95% Confidence Interval)^a^	*p* Value
Phases 5 to 9 Combined		Outcome: Incident physician-diagnosed diabetes	
No	19,767 (274)^b^	1.00	
Yes	569 (20)^b^	2.34 (1.46, 3.75)	<.0001
Phases 3 to 9 Combined		Outcome: Incident study screen-detected diabetes	
No	20,060 (337)^b^	1.00	
Yes	569 (9)^b^	.88 (.45, 1.72)	.70

a Odds ratios are from age-, sex-, and ethnicity-adjusted multilevel logistic regression pooling person-observations over study phases with generalized estimation equation. Diabetes is considered only at first occurrence and coded as missing value at subsequent phases.

b The analysis is based on 5978 participants, of which 294 had incident physician-diagnosed diabetes and 346 incident screen-detected diabetes.

**Table 4:** Longitudinal Association Between Status of Antidepressant Use and Subsequent Change in Fasting and 2-Hour Postload Glucose Levels Among Participants Without Physician-Diagnosed Diabetes

Data Cycles 1 to 3 Combined	Number of Observations^a^	Mean (SD), mmol/L	Mean (95% CI) Difference, mmol/L^b^	*p* Value
Antidepressant Use		Outcome: Subsequent change in fasting glucose		
No	12,295	.0 (.7)	.0 (Ref)	
Yes	285	.1 (.7)	.1 (−.0, .1)	.11
Exposure to Antidepressant Use				
No	12,197	.0 (.7)	.0 (Ref)	
1 examination	300	.1 (.7)	.1 (.0, .1)	.02
2 examinations	65	−.0 (.6)	−.0 (−.2, .1)	.58
3 examinations	18	−.0 (.6)	−.0 (−.3, .3)	.81
Antidepressant Use		Outcome: Subsequent change in 2-hour postload glucose		
No	11,123	.6 (1.9)	.0 (Ref)	
Yes	234	.5 (1.7)	−.0 (−.2, .2)	.87
Exposure to Antidepressant Use				
No	11,047	.6 (1.9)	.0 (Ref)	
1 examination	250	.6 (1.6)	−.0 (−.2, .2)	.93
2 examinations	50	.3 (1.8)	−.2 (−.7, .3)	.46
3 examinations	10	.6 (2.4)	.0 (−1.1, 1.1)	.98

Data cycle 1 is from phase 3 (1991/1993) to phase 5 (1997/1999); data cycle 2 from phase 5 (1997/1999) to phase 7 (2003/2004); and data cycle 3 from phase 7 (2003/2004) to phase 9 (2008/2009). CI, confidence interval; Ref, reference group; SD, standard deviation.

a Total number of participants 5487 in analysis of fasting glucose and 4991 in analysis of postload glucose.

b From age-, sex-, and ethnicity-adjusted multilevel linear regression pooling person-observations over study phases with generalized estimation equation.
