# Supplementary material for: Antidepressant Medication Use and Risk of Hyperglycemia and Diabetes Mellitus—A Noncausal Association?
Source: Biol Psychiatry. 2011 Nov 15;70(10):978–84. doi: 10.1016/j.biopsych.2011.07.008 (PMC3202626; doi:10.1016/j.biopsych.2011.07.008)
Supplement: Supplement 1 [file mmc1.pdf]

Antidepressant Medication Use and Risk of Hyperglycaemia and Diabetes Mellitus — A Non-causal Association?

Supplemental Information

Contents

Statistical Analysis: Multilevel Modeling 2

Table S1. Cross-sectional Association Between Status of Antidepressant Use and Diabetes Mellitus by Method of Detection 4

Table S2. Longitudinal Association Between Status of Antidepressant Use and Subsequent Change in Fasting and 2-h Postload Glucose Levels Among Participants without "Physician Clinically Diagnosed" Diabetes 5

Table S3. Multivariable Adjusted Associations of Antidepressant Use with Diabetes and Change in Fasting and 2-h Postload Glucose Levels 6

Table S4. Complete Case Analysis of Age-, Sex-, and Ethnicity-Adjusted Associations of Antidepressant Use with Subsequent Change in Fasting and 2-h Postload Glucose Levels Among Participants without "Physician-diagnosed" Diabetes 7

Figure S1. Study Flow Diagram for Reverse Causation Analysis 8

Statistical Analysis: Multilevel Modeling

In multilevel analyses, we structured data so that each participant contributed 2 to 4 person-observations, depending on the number of study phases for which data for the participant were available. Thus, the availability of information on antidepressant medication and the diabetes status of each participant could vary over the four measurements.

The cross-sectional associations between antidepressant use and "physician-diagnosed" and "study screen-detected" diabetes across phases 5 to 9 (Table 3 in main article) were analyzed using multilevel logistic regression by the generalized estimating equations (GEE) method using ‘xtgee’ procedure in STATA 11.1 software with binomial logit link function, the participant being the clustering factor. This method takes into account the non-independence of repeated measurements (person-observations) of the same individual and gives a combined estimate for the following two sets of associations:

- Antidepressant use at phase 5 and incident "physician-diagnosed diabetes" at phase 5
- Antidepressant use at phase 7 and incident "physician-diagnosed diabetes" at phase 7
- Antidepressant use at phase 9 and incident "physician-diagnosed diabetes" at phase 9

and

- Antidepressant use at phase 5 and incident "study screen-detected diabetes" at phase 5
- Antidepressant use at phase 7 and incident "study screen-detected diabetes" at phase 7
- Antidepressant use at phase 9 and incident "study screen-detected diabetes" at phase 9

To assess the association between antidepressant use and subsequent change in glucose levels (Table 4, predictor “Antidepressant use” in main article), we performed multilevel linear regression analyses, with GEE (procedure xtgee, options family (normal) link (identity)) and participant as the cluster, to obtain a combined estimate for the following associations:

- Antidepressant use at phase 3 predicting change in fasting glucose between phases 3 and 5
- Antidepressant use at phase 5 predicting change in fasting glucose between phases 5 and 7
- Antidepressant use at phase 7 predicting change in fasting glucose between phases 7 and 9

and

- Antidepressant use at phase 3 predicting change in postload glucose between phases 3 and 5
- Antidepressant use at phase 5 predicting change in postload glucose between phases 5 and 7
- Antidepressant use at phase 7 predicting change in postload glucose between phases 7 and 9

We ran a corresponding analysis for the association between length of exposure to antidepressant use (defined as the number of times reported antidepressant use at the current and preceding clinical examinations) and subsequent change in glucose levels (Table 4, predictor "Exposure to antidepressant use" in main article):

- Cumulative exposure to antidepressant use by phase 3 predicting change in fasting glucose between phases 3 and 5
- Cumulative exposure to antidepressant use by phase 5 predicting change in fasting glucose between phases 5 and 7
- Cumulative exposure to antidepressant use by phase 7 predicting change in fasting glucose between phases 7 and 9

and

- Cumulative exposure to antidepressant use by phase 3 predicting change in postload glucose between phases 3 and 5
- Cumulative exposure to antidepressant use by phase 5 predicting change in postload glucose between phases 5 and 7
- Cumulative exposure to antidepressant use by phase 7 predicting change in postload glucose between phases 7 and 9

To minimize confounding arising from differences in time-independent characteristics between individuals, we repeated these analyses applying within-participant analysis of changes in glucose levels using random-intercept multilevel linear regression with the fixed-effect estimator ('xtreg' procedure with fixed-effect option in STATA). This analysis is based only on within-individual variance in antidepressant use and glucose levels. As reported in the main article, the within-individual analysis led essentially to the same conclusions as the analysis presented in Table 4.

Table S1. Cross-sectional Association Between Status of Antidepressant Use and Diabetes Mellitus by Method of Detection[a].

**Phase**<br>**Antidepressant use**	**No. of participants**<br>**(No. of diabetes cases)**	**Odds ratio**<br>**(95% CI)**	***P*-value**
Phase 5		Outcome: Incident "physician-diagnosed" diabetes	
No	5,154 (38)	1.00	
Yes	136 (1)	1.06 (0.14, 7.80)	0.96
Phase 7			
No	4,515 (85)	1.00	
Yes	148 (7)	2.78 (1.25, 6.19)	0.01
Phase 9			
No	4,473 (151)	1.00	
Yes	190 (12)	2.00 (1.08, 3.70)	0.03
Phases 5 to 9 combined†			
No	19,767 (274)‡	1.00	
Yes	569 (20) ‡	2.34 (1.46, 3.75)	<0.0001
Phase 5		Outcome: Incident "study screen-detected" diabetes	
No	5154 (73)	1.00	
Yes	136 (2)	1.11 (0.27, 4.61)	0.89
Phase 7			
No	4,515 (132)	1.00	
Yes	148 (3)	0.76 (0.24, 2.42)	0.64
Phase 9			
No	4,473 (132)	1.00	
Yes	190 (4)	0.78 (0.28, 2.13)	0.63
Phases 3 to 9 combined†			
No	20,060 (337)‡	1.00	
Yes	569 (9)‡	0.88 (0.45, 1.72)	0.70

[a]Age-, sex- and ethnicity-adjusted odds ratios are from multilevel logistic regression with generalized estimating equations method. Diabetes was considered only at first occurrence and coded as missing value at subsequent phases.

†This result is also reported in Table 3 of the main article.

‡The analysis is based on 5,978 participants of whom 294 had incident clinically diagnosed diabetes and 346 incident screen-detected diabetes.

CI, confidence interval.

Table S2. Longitudinal Association Between Status of Antidepressant Use and Subsequent Change in Fasting and 2-h Postload Glucose Levels Among Participants without "Physician Clinically Diagnosed" Diabetes.

**Data cycle** **Antidepressant use**	**No. of participants**	**Mean (SD), mmol/L**	**Mean (95% CI) difference, mmol/L***	***P*-value**
Data cycle 1		Outcome: Subsequent change in fasting glucose		
No	4,727	-0.1 (0.6)	0.0 (Ref)	
Yes	69	-0.1 (0.5)	0.0 (-0.1, 0.2)	0.94
Data cycle 2				
No	3,810	0.2 (0.7)	0.0 (Ref)	
Yes	94	0.3 (0.6)	0.1 (-0.0, 0.2)	0.17
Data cycle 3				
No	3,289	-0.1 (0.6)	0.0 (Ref)	
Yes	104	-0.1 (0.8)	0.0 (-0.1, 0.1)	0.64
Data cycles 1 to 3 combined†				
No	12,295‡	0.0 (0.7)	0.0 (Ref)	
Yes	285‡	0.1 (0.7)	0.1 (-0.0, 0.1)	0.11
Data cycle 1		Outcome: Subsequent change in 2-hour postload glucose		
No	4,436	0.8 (1.8)	0.0 (Ref)	
Yes	63	0.6 (1.7)	-0.2 (-0.6, 0.3)	0.51
Data cycle 2				
No	3,464	0.5 (1.9)	0.0 (Ref)	
Yes	77	0.5 (1.5)	0.0 (-0.4, 0.5)	0.88
Data cycle 3				
No	2,912	0.5 (1.9)	0.0 (Ref)	
Yes	80	0.6 (1.8)	0.2 (-0.2, 0.6)	0.43
Data cycles 1 to 3 combined†				
No	11,123‡	0.6 (1.9)	0.0 (Ref)	
Yes	234‡	0.5 (1.7)	-0.0 (-0.2, 0.2)	0.87

Data cycle 1 is from Phase 3 (1991/1993) to Phase 5 (1997/1999); Data cycle 2 from Phase 5 (1997/1999) to Phase 7 (2003/2004); and Data cycle 3 from Phase 7 (2003/2004) to Phase 9 (2008/2009).
*Multilevel linear regression with generalized estimating equations adjusted for age, sex, and ethnicity.
†This result is also reported in Table 4 of the main article.
‡Number of observations. Total number of participants 5,487 in analysis of fasting glucose and 4,991 in analysis of postload glucose.
CI, confidence interval.

Table S3. Multivariable[a] Adjusted Associations of Antidepressant Use with Diabetes and Change in Fasting and 2-h Postload Glucose Levels.

**Exposure variable**	**Outcome variable**	**No. of participants (observations)**	**Odds ratio (95% CI)**	**Mean (95% CI) difference, mmol/L**	***P*-value**
Antidepressant use at phase 3	Incident "physician-diagnosed" diabetes at phases 5 to 9	5804	2.84 (1.45, 5.54)		0.002
Antidepressant use at phase 3	Incident "screen-detected" diabetes at phases 5 to 9	5804	1.11 (0.48, 2.61)		0.80
Antidepressant use at phases 5, 7, and 9	Incident " physician-diagnosed" diabetes at phases 5, 7, and 9	5949 (15332)	2.34 (1.20, 4.53)		0.01
Antidepressant use at phases 5, 7, and 9	Incident "screen-detected" diabetes at phases 5, 7 and 9	5949 (15346)	0.63 (0.25, 1.57)		0.32
Antidepressant use at phases 3, 5 and 7	Change in fasting glucose from phase 3 to 5, 5 to 7 and 7 to 9	5430 (11035)		0.0 (-0.0, 0.1)	0.49
Antidepressant use at phases 3 5, and 7	Change in postload glucose from phase 3 to 5, 5 to 7 and 7 to 9	4948 (10060)		-0.1 (-0.3, 0.2)	0.67
Exposure to antidepressant use at phases 3, 5, and 7	Change in fasting glucose from phase 3 to 5, 5 to 7 and 7 to 9	5430 (11035)		0.0 (-0.0, 0.0)	0.94
Exposure to antidepressant use at phases 3, 5, and 7	Change in postload glucose from phase 3 to 5, 5 to 7 and 7 to 9	4948 (10060)		-0.1 (-0.2, 0.1)	0.37

[a]Adjusted for age, sex, ethnicity, occupational position, body mass index, waist, systolic blood pressure, HDL-cholesterol, triglycerides, antihypertensive medication, lipid-lowering medication, smoking, alcohol consumption, and physical activity at the same phases as the exposure variable (except for ethnicity and sex). Age-, sex-, and ethnicity-adjusted associations are reported in Tables 2, 3, and 4 of the main article.

CI, confidence interval; HDL, high-density-lipoprotein.

Table S4. Complete Case Analysis of Age-, Sex-, and Ethnicity-Adjusted Associations of Antidepressant Use with Subsequent Change in Fasting and 2-h Postload Glucose Levels Among Participants without "Physician-diagnosed" Diabetes[a].

Exposure variable	Outcome variable	No. of participants (observations)	Mean (95% CI) difference, mmol/L	*P*-value
Antidepressant use at phases 3, 5, and 7	Change in fasting glucose from phase 3 to 5, 5 to 7 and 7 to 9	3973 (10572)	0.1 (-0.0, 0.1)	0.07
Antidepressant use at phases 3, 5, and 7	Change in postload glucose from phase 3 to 5, 5 to 7 and 7 to 9	3698 (9625)	0.0 (-0.2, 0.3)	0.69
Exposure to antidepressant use at phases 3, 5, and 7	Change in fasting glucose from phase 3 to 5, 5 to 7 and 7 to 9	3973 (10572)	0.0 (-0.0, 0.1)	0.18
Exposure to antidepressant use at phases 3, 5 and 7	Change in postload glucose from phase 3 to 5, 5 to 7 and 7 to 9	3698 (9625)	0.0 (-0.1, 0.1)	0.98

[a]Results based on all available person-observations in the cohort of participants without "physician-diagnosed" diabetes are reported in Table 4 of the main article.

CI, confidence interval.

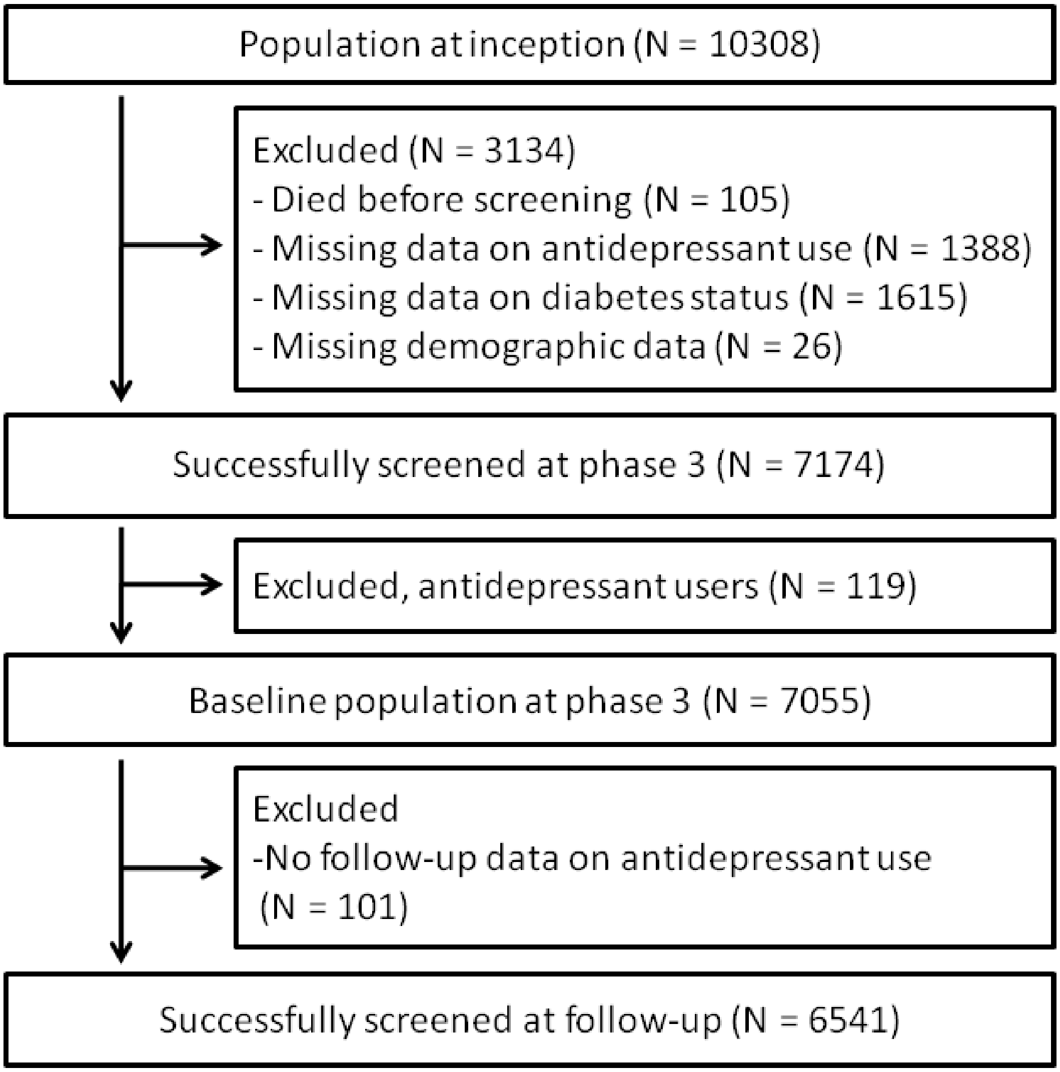

Figure S1. Study Flow Diagram for Reverse Causation Analysis
